# Prevalence and Geographic Distribution Pattern of Asthma in Tehran by ECRHS

**Published:** 2016

**Authors:** Hooman Sharifi, Mostafa Ghanei, Makan Sadr, Habib Emami, Atefeh Fakharian, Zahra Hessami, Mahshid Aryanpur, Hamidreza Jamaati, Mohammad Reza Masjedi

**Affiliations:** 1 Tobacco Prevention and Control Research Center, National Research Institute of Tuberculosis and Lung Diseases (NRITLD), Shahid Beheshti University of Medical Sciences, Tehran, Iran; 2 Baqiyatallah Research Center of Gastroentrology and Liver Diseases, Baqiyatallah University of Medical Sciences, Tehran, Iran; 3 Tracheal Diseases Research Center, NRITLD, Shahid Beheshti University of Medical Sciences, Tehran, Iran; 4 Chronic Respiratory Diseases Research Center, NRITLD, Shahid Beheshti University of Medical Sciences, Tehran, Iran; 5 Tobacco Control Research Center, Iranian Anti Tobacco Association, Tehran, Iran.

**Keywords:** Asthma, Asthma-like symptoms, Prevalence

## Abstract

**Background:**

Asthma, involving 5–10% of global population, has a heterogeneous distribution in the community regardless of age and its prevalence and incidence tend to grow worldwide as reported by many recent epidemiologic surveys.

**Materials and Methods:**

The present study aimed to survey the prevalence of asthma symptoms in Tehran as the first attempt in terms of situation analysis of the disease in the Iranian society by using relevant parts of the European Community Respiratory Health Survey (ECRHS) questionnaire.

**Results:**

Wheezing was reported in 48% of males and 34% of females in the age range of 20–44 years, around 50% of which was associated with breathlessness or cold., the people who answered “yes” to the questions 1 (wheezing), 4 (coughing), 5 (asthma history) or 7 (nasal allergy) were totally 211 among whom 124 (58.8%) were males and the rest (41.2%) were females.

**Conclusion:**

Asthma symptoms decrease the quality of life and impose high costs on the healthcare system in many countries. A low rate of participants had been informed about their asthma by physicians and not all of them were taking medications. Risk factor analysis and control is strongly advised in order to lessen the prevalence of the disease and symptoms. Air pollution, smoking, unhealthy life style and many personal and social factors need to be assessed and eliminated. It seems that a- second phase- ECRH survey should be conducted to assess the situation of asthma through population of Tehran.

## INTRODUCTION

Bronchial asthma is known as a complex chronic inflammatory disease which is usually characterized by reversible airway obstruction and hyper-reactivity. Asthma is often diagnosed with symptoms such as wheezing, nocturnal or early morning cough, and shortness of breath by physicians in community setting (1–6). This disease has a heterogeneous distribution in the community irrespective of age and its prevalence and incidence tend to grow worldwide as reported by many recent epidemiologic surveys (1, 7). The incidence of asthma cannot be determined precisely, mainly due to the lack of a general gold standard for asthma definition and causes (6, 8). An observable growth in asthma prevalence in the recent years has been a strong motivation to start comprehensive global studies in this regard (9, 10). This rapidly increased prevalence is not caused by a single factor like genetics, age, or air pollution. Thus, many factors are to be evaluated in this disease (11). A trend study in Italy by de Marco and colleagues shows a growing prevalence of asthma and allergic rhinitis as well as asthma-like symptoms from 1991 to 2010 and the authors point to a 38% increase in asthma prevalence during 20 years (12). Similar attempts in Iceland by Sigurkarlsson et al. shows three-fold increase in the prevalence of asthma attacks and use of anti-asthmatic drugs between 1990 and 2007 in the young population (13). This condition has created a significant burden on the healthcare system due to increased morbidities and reduced quality of life (14, 15). Population-based epidemiologic studies are ongoing using several types of questionnaires focusing on clinical symptoms and past medical history beside environmental and individual risk factors. Based on global guidelines, it is inevitable to suspect asthma in case of respiratory symptoms including chronic cough, wheeze, dyspnea and chest tightness in addition to bronchial hypersensitivity (16). The most renown surveys that used questionnaires are the International Study of Asthma and Allergies in Childhood and the European Community Respiratory Health Survey (ECRHS)(17), among which the latter was successfully followed by the present study. The ECRHS was the first study to assess the situation of asthma in adults in 25 countries in three phases evaluating geographical prevalence, risk factors, treatment and follow up of asthma (9, 17, 18). The questionnaire used in ECRHS is now one of the most popular instruments for epidemiologic studies due to its validity and acceptability. It contains 10 simple and specific questions used perfectly in large-scale surveys on asthma (1, 19) although the subjective character of the questionnaire limits the accuracy of asthma detection and may overestimate the prevalence of the disease (20). However, the ECRHS questionnaire has been the most perfect tool to obtain data in this matter so far, which could be used in the Middle East where the data regarding asthma prevalence are scarce (1).

There have been other studies working on asthma in children. To our knowledge, no population-based study has explored the prevalence of adult asthma in Tehran. Accordingly, the present study aimed to assess the prevalence of asthma in Tehran as the first attempt in situation analysis of the disease in an Iranian society by using relevant parts of the ECRHS questionnaire.

## MATERIALS AND METHODS

**Population and sampling strategy:** The study was done through a cross-sectional design to assess the frequency of asthma and wheezing in Tehran with 8.1 million population. We used stratified cluster sampling considering urban regions and the density of population all over the city.

**Sample Size:** To obtain a study power of at least 80% in addition to the effect size of l.5, and a response rate of 60%, the sample size was calculated to be 961 participants to answer “yes” to the questions 3, 5 or 6 of ECRHS questionnaire. We needed to enroll 3,366 people to obtain accurate results.

**Sampling**: As pointed out before, the design for sampling was a stratified cluster strategy using proportional allocation within strata. The targeted population was all residents over 18 years of age who lived in the studied clusters of Tehran in 2013. The participants were recruited in two ways in terms of age allocation. First, we divided them into three age groups including <20, 20–39, and > 40 years in order to evaluate demographics, the frequency of relevant symptoms and also the medical history of people. Then, the participants were studied if they were between 20 and 44 years considering the standards which are compulsory to observe in ECRHS screening.

The stratification was done considering the 22 municipal districts of Tehran. Appreciating the population density in different 22 districts of Tehran, the appropriate number of clusters was weighted according to each district. This number was also affected by the total sample size, mean number of household members, and logistical facilities for subject enumeration, transport, and examination. There were three-member teams to refer the clusters in order to obtain data. Two interviewers, a man and a woman dressed in white medical coats, in addition to a driver were recruited. The interviewing team approached the index household specified via a random selection of clusters, and continued the enumeration in 10 neighboring households in a systematic manner by proceeding in a clock-wise direction. The interviewers were advised to try the Kish Grid or method to choose the right participant(s) when there was more than one person in the indexed household. The named method is simply a table of numbers which is used to find the number of residents in the household. Then, a randomly selected number would determine the person who is the one to recruit.

**Definition:** Asthma is a chronic inflammatory disorder associated with variable airflow obstruction and bronchial hyper-responsiveness. It presents with recurrent episodes of wheeze, cough, shortness of breath and chest tightness (6).

**Examination Protocol:** A comprehensive ECRHS questionnaire was used through a broad survey and the present study was part of it. All questionnaires were filled out by interviewers. Respiratory symptoms, health status, activity limitation, and risk factor exposure were the items to assess among which symptoms and signs of asthma, especially wheezing in addition to relevant medical history were the most focused ones. The core questionnaire was developed using preexisting validated questionnaire that had already been used in multinational studies.

**Statistical Analysis:** The present study used SPSS 21 for windows to gather the data and analyze them considering the 95% confidence interval while defining the significance <0.05 and type 1 error equal to 0.05. The frequencies were reported through central statistical tendency indices using t-test and chi-square test. More details in terms of the materials and methods of this study have already been published elsewhere (21).

## RESULTS

Totally, 3,366 subjects enrolled the survey by answering the ECRHS questionnaire amongst them 1,755 (52.2%) were males and 1,611 (47.8%) females (Table 1).

**Table 1.** Demographic characteristics of participants by age

**Variable**	**<20 yrs. n=235**	**20–39 yrs. n=2682**	**40 yrs. or more n=388**	**Total**
Male gender, n(%)	164 (69.8%)	1383 (51.6%)	168 (43.4%)	1715
Mean age years ±SD	17.85±2.07	29.68±5.40	45.07±8.49	30.65±8.32

The current age was not reported by 61 participants who comprised missing cases in this regard. The mean age was 30.65 ± 8.32 years. The majority of participants were between 20 and 39 years of age (81.2%) (Table 1).

**Figure 1.**
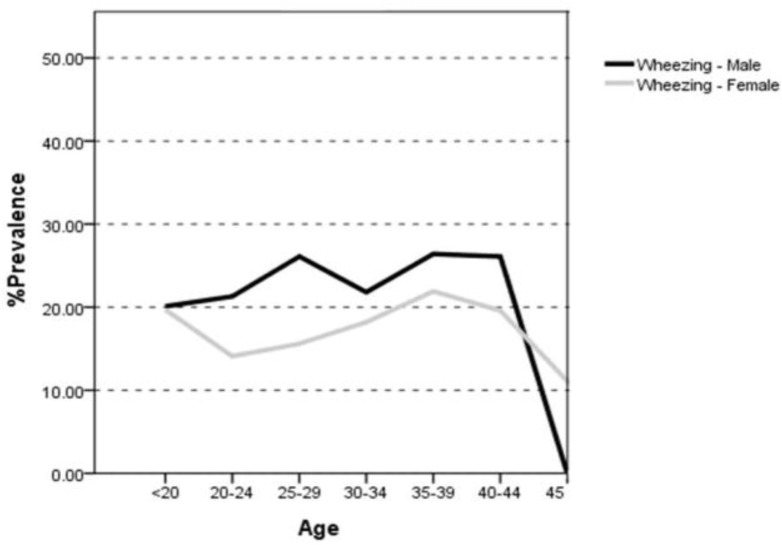
The trend of wheezing prevalence in males and females according to their age

As reported in Table 2, patients’ symptoms were asked in three different age groups. Wheezing was found in 37.5% of people <20 years of age and 45% of older people. Chest tightness was more frequent in 20–39 year olds (22.3%); 3.3% of the participants had been informed to have asthma by physicians before and 2.3% of them were using asthma medications. The total prevalence rate of nasal allergy (including hay fever) was 21.8% in all age groups which was a bit less frequent in ≥ 40 year olds. Men had higher rate of wheezing in all age groups than women as can be seen in Figure 1.

**Table 2.** Symptoms according to the ECRHS screening questionnaire in participants, by age

**ECRHS screening questionnaire symptoms within the last 12 months, n (%)**	**<20 yr. n=235**	**20–39 yr. n=2682**	**40 yr. or more n=388**	**Total n=3366**
Wheezing/whistling	47 (20.0%)	560 (20.9%)	83 (21.4%)	700 (20.8%)
Wheezing with breathlessness	10 (4.3%)	173 (6.5%)	40 (10.3%)	224 (6.7%)
Wheezing without a cold	31 (13.2%)	355 (13.2%)	57 (14.7%)	448 (13.3%)
Chest tightness	46 (19.6%)	597 (22.3%)	77 (19.8%)	743 (22.1%)
Have you ever experienced shortness of breath?	66 (28.1%)	713 (26.6%)	113 (29.1%)	899 (26.7%)
Attack of coughing	91 (38.7%)	1002 (37.4%)	128 (33.0%)	1241 (36.9%)
Are you informed by the physician that you got asthma?	7 (3.0%)	84 (3.1%)	16 (4.1%)	111 (3.3%)
Currently taking asthma medications	7 (3.0%)	61 (2.3%)	8 (2.1%)	79 (2.3%)
Nasal allergies (including hay fever)	52 (22.1%)	611 (22.8%)	67 (17.3%)	733 (21.8%)

Out of 3,305 responders, 211 said “yes” to the questions Q1 “wheezing”, Q4 “coughing”, Q5 “asthma”, or Q7 “nasal allergies” (6.3%) but 1,316 (39.1%) said “no” to all questions (Table 3).

**Table 3.** Characteristics of those responding “YES” to Q1 “wheezing”, Q4 “coughing”, Q5 “asthma”, or Q7 “nasal allergies” according to the ECRHS screening questionnaire (n = 211)

**Variable**	**Male (n=124)**	**Female (n=87)**	**Total (n=211)**	**P-value**
**Age in years, mean (SD)**	29.33±7.77	31.43±7.690	30.33±7.75	<0.001
**Age Groups**				
<20	13 (9.6%)	3 (4.9%)	16 (7.4%)	
20–39	89 (80.1%)	82 (81.7%)	171 (80.9%)	<0.001
40 or more	13 (8.3%)	8 (12.5%)	21 (10.3%)	
Unknown	2 (1.9%)	1 (1.0%)	3 (1.5%)	
**ECRHS screening questionnaire symptoms within the last 12 months, n (%)**							
Wheezing/whistling	49 (44.5%)	33 (34.4%)	82 (39.7%)	<0.001
Have you ever experienced shortness of breath?	38 (34.2%)	43 (42.9%)	81 (38.3%)	<0.001
Attack of coughing	80 (72.2%)	68 (68.4%)	148 (70.4%)	0.076
Are you informed by the physician that you got asthma?	7 (6.3%)	6 (6.3%)	13 (6.3%)	0.942
Currently taking asthma medications	5 (4.4%)	4 (4.3%)	9 (4.4%)	0.905
Nasal allergies (including hay fever)	43 (39.2%)	44 (44.3%)	87 (41.6%)	0.030

**Figure 2.**
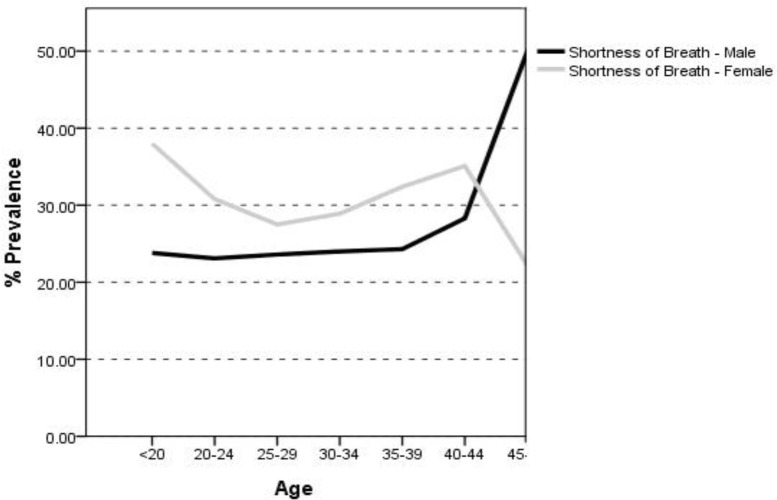
The trend of breathlessness prevalence in males and females according to their age

In terms of breathlessness, both males and females showed a slow process in its occurrence as the age raised (Figure 2).

As seen in Table 3, the people who answered “yes” to the questions 1 (wheezing), 4 (coughing), 5 (asthma history) or 7 (nasal allergy) were totally 211 among whom 124 (58.8%) were males and the rest (41.2%) were females. The mean age was significantly different between males and females (P<0.001).

Based on the protocol of ECRHS, people between 20–44 years of age were included in the present study. Thus, as presented in Table 4, of 2,984 individuals 51% were males and 49% were females in the selected age range. Wheezing was reported in 48% of males and 34% of females around a half of which was with breathlessness or cold. Wheezing and the history of dyspnea were the significantly different items with regard to participants’ gender.

**Table 4.** Symptoms according to the ECRHS screening questionnaire in participants 20–44 yr, by gender

**ECRHS screening questionnaire symptoms within the last 12 months, n (%)**	**20–44 yr. Male n=1521**	**20–44 yr. Female n=1463**	**P-value**
Wheezing/whistling	366 (24.1%)	262 (17.9%)	<0.001
Wheezing with breathlessness	123 (8.1%)	84 (5.7%)	0.012
Wheezing without a cold	247 (16.2%)	153 (10.5%)	<0.001
Chest tightness	355 (23.3%)	300 (20.5%)	0.060
Have you ever experienced shortness of breath?	367 (24.1%)	444 (30.5%)	<0.001
Attack of coughing	584 (38.4%)	522 (35.7%)	0.129
Are you informed by the physician that you got asthma?	47 (3.1%)	48 (3.3%)	0.754
Currently taking asthma medications	36 (2.4%)	31 (2.1%)	0.623
Nasal allergies (including hay fever)	323 (21.2%)	350 (23.9%)	0.079

## DISCUSSION

In this survey, we tried to assess the frequency and other aspects of asthma symptoms among studied clusters of population in Tehran who were between 20 to 44 years old. Wheeze, cough, dyspnea and chest discomfort were the most common symptoms to ask as well as history of asthma and medication use. A low rate of participants had been informed about their asthma by physicians and not all of them were taking medications. Wheezing and cough had around 40% prevalence while chest tightness and dyspnea were less frequent. People older than 40 years had the highest rate of wheeze whilst those younger than 40 years old had more cough. Thus, wheezing and dyspnea were more commonly found in the elderly although not significant. To continue the ECRHS protocol, males between 20–44 yrs. had more wheezing than females and only wheezing and dyspnea were significantly different according to participants’ sex in this age range. But a Spanish team in 2010 showed that teenager males and active smoking had no association with the prevalence of asthma but female gender was associated with wheezing and nocturnal dry cough (22). The same was reported in Belgrade, Serbia (23).

Asthma affects 5–10% of the world’s population. Its severity is assessed by pulmonary function test, symptom frequencies and exacerbation rate as well as rescue inhaler use (24). It is mainly a childhood disease although it is too hard to know its precise definition and prevalence in adults (25). A report by Chuchalin et al. in Russia showed 25.7% prevalence of asthma symptoms and 6.9% asthma prevalence based on patients’ self-reported diagnosis (26). They used GARD questionnaire for chronic respiratory disease prevalence instead of ECRHS.

The quality of life has been raised in the recent decades and chronic respiratory disease including asthma have obviously been focused in this regard. One of the relevant studies in this regard was done by Luskin et al. to assess the reduction in quality of life based on asthma severity (27). We just assessed the frequency of asthma symptoms but many studies have reported triggers of the disease. For instance, Young and colleagues found associations between air pollution and symptoms including wheezing and cough in addition to asthma incidence in American women (28).

A survey by Aumann et al, in Germany was conducted to assess the situation and cost of asthma through a systematic review (29). They reported 4–6% prevalence of asthma in adults and estimated the costs to be between 700 million and 1.4 billion Euros per year. Boys and adult females were the most commonly involved population with asthma, similar to many reports by different authors including the present study. The prevalence of asthma is usually a result of many factors and interestingly, chronic obstructive pulmonary disease would be a predisposing factor or a consequence of asthma. Age-specific prevalence of confirmed chronic obstructive pulmonary disease was higher among active asthmatic adults when compared with non-active cases (30, 31).

Asthma control seems very difficult as even European surveys showed uncontrolled or poorly controlled asthma cases (32). A surprising study by Hoppin et al, in two states in the United States reported lower rates of respiratory diseases such as asthma, chronic bronchitis, and emphysema and higher rate of respiratory symptoms such as wheezing or cough among farmers and their families compared to the general population between 2005 and 2010 (33). This was after controlling for the effects of smoking, BMI, and population characteristics. Uğurlu et al. published their survey using ECRHS method aiming to find much frequent asthma cases or asthma symptoms in rural regions but could not find a significant difference between rural and urban areas in Turkey (34). Another similar study in Turkey by Talay et al. showed a correlation between socioeconomic level and increased risk of asthma and allergic rhinitis in addition to some asthma symptoms, especially in smokers (35). Mahboub et al, in the United Arab Emirates conducted a survey using ECRHS and concluded that asthma symptoms were common in their country with 8–10% prevalence rate, like any other region in the world (1). They found no difference between the sexes in this matter, as also noted in our study. Some researchers have even aimed to find genetic effects on the frequency and distribution of asthma symptoms and disease to find polymorphisms and probable mutations in some target genes (36).

In conclusion, asthma symptoms compromise the quality of life besides directing high costs on the healthcare system in many countries and would be too hard to control, especially in developing countries with limited health budget. Thus, risk factor analysis and control is strongly advised in order to lessen the prevalence of the disease and symptoms. Air pollution, smoking, imperfect life style and many personal and social factors need to be assessed and eliminated.
